# Characterization of Plasma Cell-Free DNA Integrity Using Droplet-Based Digital PCR: Toward the Development of Circulating Tumor DNA-Dedicated Assays

**DOI:** 10.3389/fonc.2021.639675

**Published:** 2021-05-06

**Authors:** Geoffroy Poulet, Fanny Garlan, Sonia Garrigou, Eleonora Zonta, Leonor Benhaim, Marie-Jennifer Carrillon, Audrey Didelot, Delphine Le Corre, Claire Mulot, Philippe Nizard, Frederic Ginot, Audrey Boutonnet-Rodat, Helene Blons, Jean-Baptiste Bachet, Julien Taïeb, Aziz Zaanan, Vanna Geromel, Laurence Pellegrina, Pierre Laurent-Puig, Shu-Fang Wang-Renault, Valerie Taly

**Affiliations:** ^1^Centre de Recherche des Cordeliers, INSERM, CNRS, Sorbonne Université, USPC, Université de Paris, Equipe labellisée Ligue Nationale Contre le Cancer, CNRS SNC 5096, Paris, France; ^2^Eurofins-Biomnis, Specialized Medical Biology Laboratory, Lyon, France; ^3^Department of Visceral and Surgical Oncology, Gustave Roussy, Villejuif, France; ^4^CIC-EC4 URC, HEGP, Hôpitaux Universitaires Paris Ouest AP-HP, Paris, France; ^5^Adelis, ID-solutions, Labège, France; ^6^Department of Oncology, European Georges-Pompidou Hospital, AP-HP, Paris Descartes University, Paris, France; ^7^Sorbonne Universités, UPMC Université, Paris, France; ^8^Department of Hepato-gastroenterology, Groupe Hospitalier Pitié Salpêtrière, Paris, France; ^9^AGEO (Association des Gastroentérologues Oncologues), Paris, France

**Keywords:** circulating tumor DNA, picoliter-droplet digital PCR, cancer biomarker, apoptosis, necrosis, DNA integrity index, circulating cell-free DNA

## Abstract

**Background:** Cellular-cell free-DNA (ccfDNA) is being explored as a diagnostic and prognostic tool for various diseases including cancer. Beyond the evaluation of the ccfDNA mutational status, its fragmentation has been investigated as a potential cancer biomarker in several studies. However, probably due to a lack of standardized procedures dedicated to preanalytical and analytical processing of plasma samples, contradictory results have been published.

**Methods:** ddPCR assays allowing the detection of *KRAS* wild-type and mutated sequences (*KRAS* p.G12V, pG12D, and pG13D) were designed to target different fragments sizes. Once validated on fragmented and non-fragmented DNA extracted from cancer cell lines, these assays were used to investigate the influence of the extraction methods on the non-mutated and mutated ccfDNA integrity reflected by the DNA integrity index (DII). The DII was then analyzed in two prospective cohorts of metastatic colorectal cancer patients (RASANC study *n* = 34; PLACOL study *n* = 12) and healthy subjects (*n* = 49).

**Results and Discussion:** Our results demonstrate that ccfDNA is highly fragmented in mCRC patients compared with healthy individuals. These results strongly suggest that the characterization of ccfDNA integrity hold great promise toward the development of a universal biomarker for the follow-up of mCRC patients. Furthermore, they support the importance of standardization of sample handling and processing in such analysis.

## Introduction

The presence of circulating cell-free DNA (ccfDNA) in body effluents has been largely described (1), and increased amounts of ccfDNA have been found in blood samples of patients affected by several disorders (2) including cancer (3, 4). The main sources of ccfDNA in healthy individuals have been described as apoptosis, active cellular release, and necrosis (5). Its integrity (defined as a metric describing size distribution) may differ depending on the involved cell-death mechanism and/or on the cell origin. In particular, it has been suggested that ccfDNA originating from tumor cells presents a different fragmentation profile than the ccfDNA originating from healthy cells (6, 7). Nevertheless, it should be taken into account that ccfDNA extracted from plasma of cancer patients is therefore composed at various proportions of tumor and non-tumor ccfDNA fractions. In recent years, the ccfDNA analysis, and more precisely its tumor fraction (ctDNA) has been largely developed, for the diagnosis, prognosis, follow-up, and treatment management of cancer patients (6, 8). Moreover, recent technological development of sensitive procedures such as droplet-based digital-PCR (ddPCR) or newly optimized next-generation sequencing (NGS) have greatly facilitated such applications (9).

The presence of ctDNA is generally assessed by the detection of tumor-specific genetic or epigenetic alterations (10–12). The quantity of ctDNA is suggested to reflect tumor burden and progression (13). In cancer, it has also been shown that disease-related cell death may lead to specific profiles of circulating nucleic acids (5). The ccfDNA integrity has thus been proposed as a potential non-invasive diagnostic biomarker especially pertinent in cancer (14, 15).

Quantitative PCR (qPCR) analysis based on the measurement of the relative amount of DNA amplicons of different sizes has long been the reference method for DNA integrity assessment (16–18). A wide range of strategies including high-sensitivity electrophoresis fragment separation (19) or NGS (20) has now been developed for such analysis. Even if the characterization of ccfDNA fragmentation profile has gained increasing interest in recent years as a cancer biomarker, contradictory results have been published (6, 16, 21). Such discrepancies could be linked with differences in the strategies used for ccfDNA fragmentation analysis. The latest findings tend to demonstrate a higher fragmentation level for ctDNA than for the non-tumor ccfDNA fraction (15, 21, 22). Using IntPlex, an optimized qPCR technique, Moulière et al. found a larger quantity of smaller fragments in plasma of colorectal cancer (CRC) patients compared with healthy subjects (23). Jiang et al. demonstrated by massive parallel sequencing that the size of ccfDNA in hepatocellular carcinoma (HCC) patients was inversely correlated with the concentrations of ctDNA in plasma (20). Comparing fragment lengths from cell-free DNA between melanoma patients (bearing *BRAF* V600E allele) and healthy subject (*BRAF* wild-type allele), Underhill et al. observed that the BRAF V600E mutant allele occurred more commonly at a shorter fragment length than the fragment length of the wild-type allele (132–145 bp vs. 165 bp, respectively) (24). Recently, using a novel technology (BIABooster system) based on electro-hydrodynamic actuation, we also suggested the pertinence of total ccfDNA quantification and size profiling for several cancers including melanoma, CRC, non-small cell lung cancer (NSCLC) and prostate cancer (19). To circumvent potential bias linked to the strategy chosen for ccfDNA integrity characterization and to increase the accuracy of such measurement, there is a growing need for highly sensitive and specific approaches.

In this article, we developed a ccfDNA integrity index (DII) dedicated-ddPCR assays and coupled it to fragment-based analysis by BIABooster system to investigate the pertinence of the characterization of total ccfDNA integrity in mCRC patients and propose its potential use as biomarker for mCRC patients. Moreover, our work also highlighted the crucial importance of the standardization of the preanalytical strategy for ccfDNA integrity investigation.

## Materials and Methods

### Patients and Study Design

Samples from two prospective cohorts of mCRC patients were included for a total of 81 samples. In the AGEO RASANC cohort, 69 patients were included with plasma samples collected in BCT Streck Tube (STRECK, Cat. N°:218997) (25). In the PLACOL cohort, 12 patients were included with plasma sampled in EDTA Tube (Greiner Bio-One International, Cat. No. 456043) (26). The basic clinical characteristics of healthy subjects and mCRC patients included in the study are summarized in Supplementary Table 1. All samples from AGEO RASANC study have been analyzed by NGS with the Panel Colon-Lung Cancer V2 (study results already published) (25). Both studies have received ethical approval from the “Ile-de-France ethics committee,” and the patients provided written informed consent. The research protocols were registered in clinicaltrials.gov (NCT02502656 and NCT01983098 for AGEO RASANC and PLACOL studies, respectively).

For both cohorts, the preanalytic conditions including blood collection tubes and plasma ccfDNA extraction kits were different. We thus added two control groups composed of samples from healthy patients that we processed with the same preanalytic conditions.

**Figure 1:**
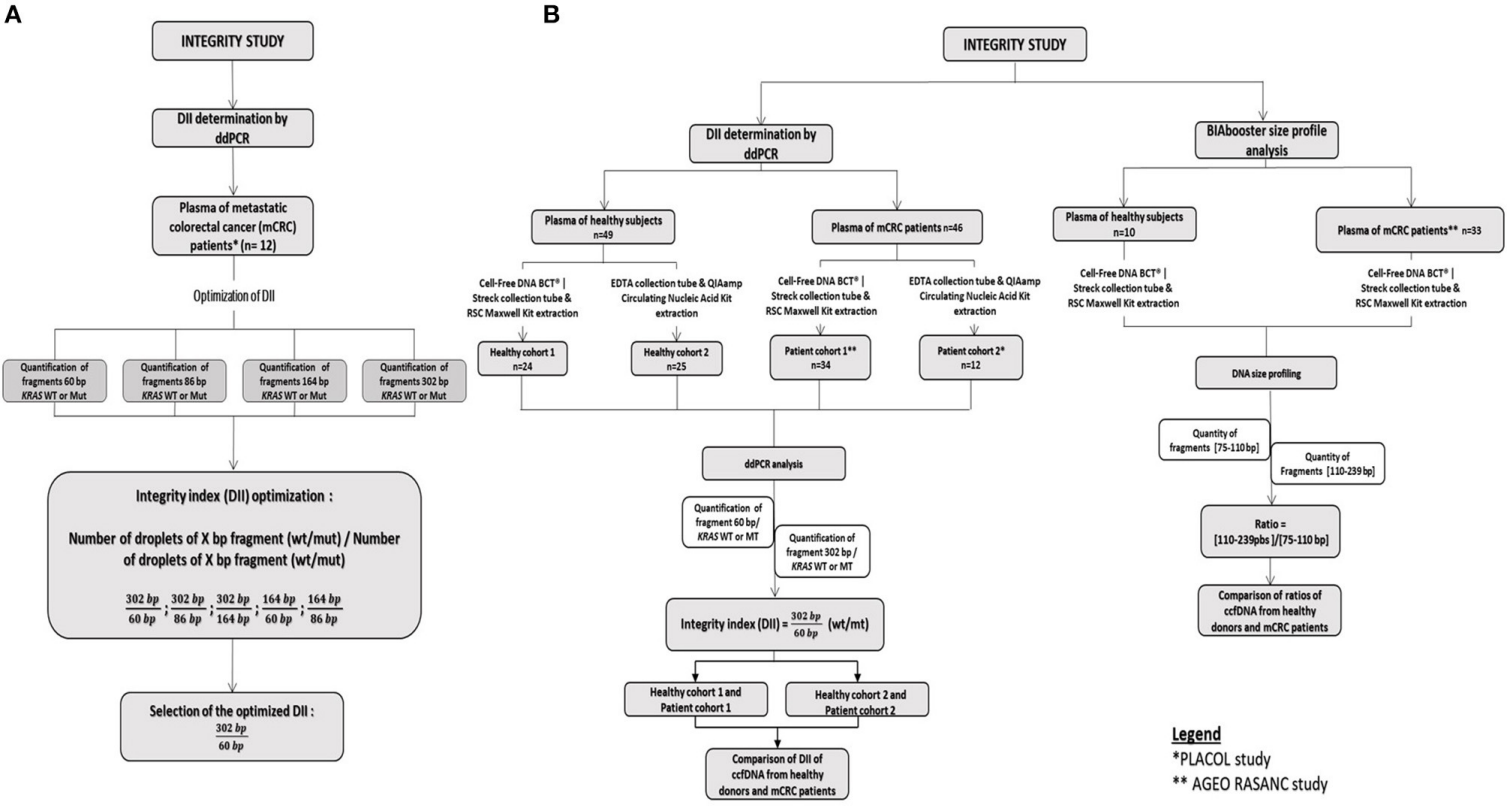
Workflow of the integrity study. **(A)** Development of the DII using the PLACOL cohort (*n* = 11 samples analyzed). DNA hasbeen quantified using, respectively, 60, 86, 164, and 302 bp amplicons. Several combinations have been tested to determine the optimal DNA integrity index. Finally, the 302/60 bp ratio turned out to be the most clinically relevant. **(B)** Samples from two prospective cohorts of patients presenting a mCRC were included, AGEO RASANC (*n* = 67) and PLACOL (*n* = 12) cohort with a total of 81 metastatic CRC samples. Circulating cell-free DNA integrity of 34 patients (AGEO RASANC cohort) and 12 patients (PLACOL cohort) was analyzed by ddPCR, and ccfDNA size profiling of 33 patients (AGEO RASANC cohort) was analyzed by BIAbooster System. As the preanalytic conditions (blood collection tubes, plasma ccfDNA extraction kits) are different for these two prospective cohorts of patients, the corresponding control groups were included; 8.5 ml blood per tube from 59 healthy subjects are used as negative controls and split into two groups (control groups A and B). Thirty-four healthy blood samples were collected in cell-free DNA BCT from Streck, and circulating DNA has been extracted with Maxwell RSC ccfDNA Plasma Kit from the plasma samples associated, and 25 blood tubes were sampled in EDTA tubes and circulating DNA purified by the QIAmp® Circulating Nucleic Acid Kit (Qiagen) from plasma samples associated. Among control A and B healthy subjects' samples, DNA integrity of 49 plasma was analyzed by droplet-based digital PCR, and ccfDNA size profiling of 10 plasma was analyzed by BIAbooster System.

The DNA integrity was evaluated by ddPCR in 46 mCRC samples and 49 healthy samples. The size profiling of ccfDNA was investigated by BIAbooster System in 33 mCRC samples and 10 healthy samples. The workflow of the study is summarized in Figure 1.

In parallel, to determine the impact of the extraction method on the ccfDNA integrity, two different ccfDNA purification kits used in PLACOL and AGEO RASANC studies (respectively, 1° QIAmp® Circulating Nucleic Acid Kit and 2° RSC Automated Maxwell) have been compared on eight healthy plasmas. The workflow of the study is listed in Supplementary Figure 1.

### Plasma ccfDNA Preparation

In the AGEO RASANC and corresponding control group, plasma from healthy individuals or mCRC patients were collected in Cell-Free DNA BCT® | Streck tubes and extracted with the use of the Maxwell RSC ccfDNA Plasma kit (Promega; called Ext.kit 1 in the manuscript) using Maxwell® RSC Instrument.

In the PLACOL study and corresponding control group, plasma from healthy individuals or mCRC patients were collected in EDTA tubes and extracted with the QIAmp® Circulating Nucleic Acid Kit (QIAGEN; called Ext.kit 2 in the manuscript) according to the manufacturer's instructions. See Supplementary Materials and Methods for full protocol description.

### Cell Line DNA Extraction, Fragmentation, and Quantification

DNA was extracted from two cell lines SW620 (mutated *KRAS* c.35G>T, p.G12V) and LoVo cell lines (mutated c.38G>A, p.G13D) using the QIAamp DNA Blood Mini Kit (QIAGEN) according to the manufacturer's instructions and eluted into 200 μl of Tris 10 mM/pH 8 before storage at −20°C.

The extracted DNA was fragmented by sonication (S220 Covaris Focused-Ultrasonicator sonicator) to obtain fragments ranging from 50 to 700 bp. The experimental parameters are described in Supplementary Table 2. The size distribution of fragmented DNA was confirmed by the miniaturized electrophoresis Caliper system, LabChip® GX/GXII Microfluidic system (Perkin-Elmer) using DNA assay 5K reagent kit (Perkin-Elmer) (see Supplementary Figure 2 for size distribution).

### Integrity Index Determination

#### Droplet-Based Digital PCR Analysis

**Figure 2:**
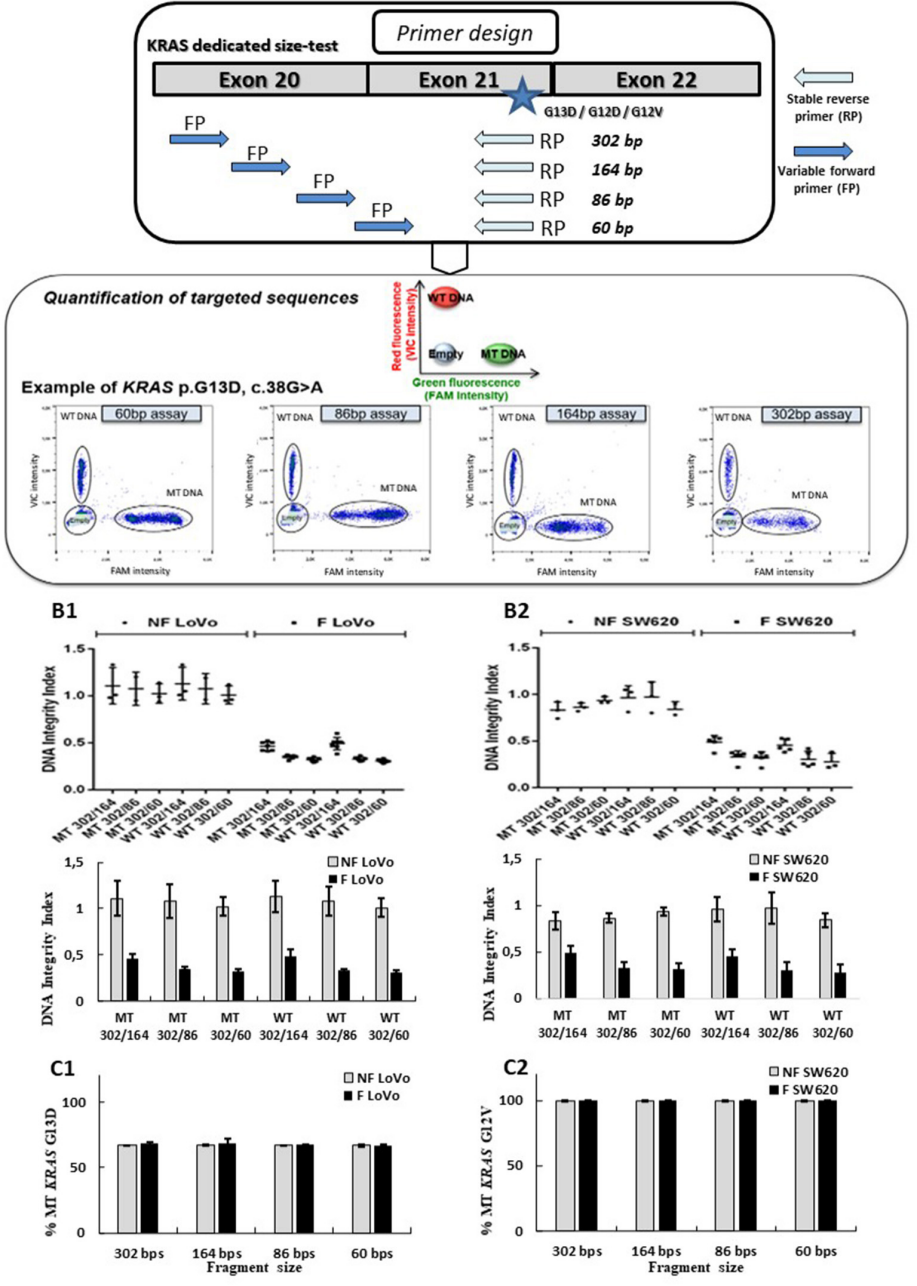
Design and validation of ddPCR assays for ccfDNA integrity analysis with the use of fragmented and non-fragmented cell line DNA. Droplet-based digital PCR duplex assays amplifying different sizes of *KRAS* wild-type (WT) and mutant (MT) fragments (60, 86, 184, and 302 bp) were designed and optimized **(A)**. The LOB for the 302-, 164-, 86-, and 60-bp *KRAS* assays were, respectively, 0, 0, 3, and 1, irrespective of the mutation status. Fragmented (F) and non-fragmented (NF) DNA from LoVo (**B1**, **C1**) and SW620 CRC line (**B2**, **C2**) were used. DNA integrity index (DII) were analyzed using 302 bp amplicon (corresponding to the long fragments) and 60, 84, and 184 bp amplicons as short fragments (**B1**, **B2**). Percentage of *KRAS*-mutated alleles for F and NF DNA of each cell lines are given in (**C1**, **C2**). Nevertheless, PCR efficiency of the primers has not been determined by qPCR for each amplicon size on fragmented and unfragmented DNA.

Droplet-based digital PCR assays targeting three frequent mutations of the *KRAS* oncogene (p.G13D, p.G12V, or p.G12D) and its wild-type sequence were designed. These assays allowed to amplify sequences of 60, 86, 164, and 302 bp for both targeted mutant (MT) and wild-type (WT) *KRAS* sequences (probe bearing a FAM or VIC fluorophore respectively) for each targeted amplicon size (see Figure 2A; Supplementary Table 3). Droplet digital PCR was carried out with the Raindrop system (Raindance Technologies, Bio-Rad) as previously described (25, 26).

#### Detailed Description of Droplet-Based Digital PCR Assays and DII Calculation

Taqman® Genotyping Master Mix (Life Technologies) at 12.5 μl was mixed with the assay solution containing: 0.75 μl of 40 mM dNTP Mix (New England BioLabs), 0.5 μl of 25 mM MgCl_2_, 1 μl 25× Droplet Stabilizer (RainDance Technologies), 1.25 μl 20× Assay Mix containing 8 μM of forward and reverse primers, 4 μM of 6-FAM and 4 μM of VIC Taqman®-labeled probes, and cell line DNA or plasma DNA to a final reaction volume of 25 μl.

All PCR assay mixes were prepared as shown in a pre-PCR room to limit risks of contamination. TaqMan® (from Life Technologies-Thermo Fisher Scientific) probes were tested. In classic TaqMan® assay, the probe bearing VIC-fluorophore (λex 538 nm/λem 554 nm) was designed to be specific to the WT allele, while the probe bearing FAM-fluorophore (λex 494 nm/λem 518 nm) was able to specifically hybridize to the mutated sequence. The emulsifications of DNA samples were generated according to manufacturer protocol (Raindance, Bio-Rad Laboratories). The emulsion was thermal cycled following the different PCR programs described in Supplementary Table 4 (PCR programs). After completion, the emulsions were either stored at 4°C or processed immediately to measure the end-point fluorescence signal from each droplet.

A limit of blank (LOB) has been calculated as described previously (1). It is defined by the frequency of positive droplets measured in genomic DNA (non-mutated) (*n* = 10 minimum). The calculated LOB was subtracted from each sample. The LOB for the 302, 164, 86, and 60 bp *KRAS* assays were, respectively, 0, 0, 3, and 1, irrespective of the mutation status. Data were analyzed according to manufacturer's instructions (Raindance Analyst Flow-Jo software). Since the number of droplets reveals the quantity of amplifiable target DNA, the fraction of amplifiable DNA in patients' samples could be determined. The number of droplets was determined using the software RainDrop Analyst (Bio-Rad Laboratories). All data were normalized to 5,000,000 droplets (corresponding to the theorical number of droplets generated by automation). A LOB correction (subtraction) has been performed to calculate the final number of positive MT droplets. Starting from the same amount of DNA, amplified fragmented copies of long and short fragments were counted to calculate the DNA Integrity Index. The PCR programs are given in Supplementary Table 4.

Starting from the same amount of DNA, DII was defined as a ratio between the number of amplified copies of long (302 bp) and “short” (60, 86, and 164 bp) DNA fragments. DIIs were calculated separately for WT and MT sequences. An example of DII calculation is as follows:

(1) $$\begin{array}{l} \left(E1\right)\;DII\;WT=\;\frac{Quantity\;of\;fluorescent\;droplets\;corresponding\;to\;302\;bp\;fragment\;\left(WT\right)\;}{Quantity\;of\;fluorescent\;droplets\;corresponding\;to\;60\;bp\;fragment\;\left(WT\right)} \end{array}$$

For samples (i.e., Placol_5 and RASANC_10) that did not present detectable 302 bp fragments for the mutated allele, the DII was evaluated as follows : DII MT < 1/[quantity of fluorescent droplets corresponding to 60 or 86 or 164 bp fragments (MT)]. These two samples were however excluded from final statistical analysis.

### BIAbooster Analysis

The size profiles and concentration analysis of ccfDNA from healthy individuals and mCRC patients were also performed using BIAbooster System (Picometrics technologies, ID-Solution) as previously described (19). The system is based on the principle of DNA fragment migration by capillary electrophoresis coupled to LED-induced fluorescence (LEDIF) detectors. It allows performing size and concentration analysis of double-stranded DNA with a sensitivity of 10 fg/μl in an operating time of 20 min. Based on the quantity of the different fragments obtained, a ratio highlighting the size distribution of the tested sample is calculated by BIAbooster technology as follows (E2):

(2) $$\begin{array}{l} \left(E2\right)\;ratio\;=\;\frac{Detected\;Quantity\;of\;fragments\;with\;a\;size\;ranging\;between\;110and\;239\;bp}{Detected\;Quantity\;of\;fragments\;with\;a\;size\;ranging\;between\;75and\;110\;bp} \end{array}$$

The description of the statistical analysis is provided in the Supplementary Materials and Methods.

## Results

### Validation of ddPCR Assays for ccfDNA Integrity Analysis Using Fragmented and Non-fragmented Cell Line DNA as a Model

Even if the characterization of ccfDNA fragmentation profile has gained increasing interest in recent years as a cancer biomarker, contradictory results have been published (6, 16, 21). Such discrepancies could be linked with differences in the strategies used for ccfDNA fragmentation analysis. The first step of this study was thus to evaluate the pertinence of ddPCR in this context using model samples containing DNA of controlled sizes.

Several Taqman® assays targeting *KRAS* sequence fragments of different lengths were designed (see Figure 2A). We used mechanically fragmented DNA (sonicated) and non-fragmented DNA from LoVo and SW620 CRC cell lines bearing WT and/or MT *KRAS* genes. Length of fragments evaluated using the miniaturized electrophoresis Caliper system, LabChip® GX/GXII Microfluidic system (see section **MATERIALS AND METHODS**), varied from 50 to 70 bp to 700 to 800 bp with higher amounts of large-size fragments (see Supplementary Figure 2). In order to determine the performance of the developed assay, we compared DII and mutant allelic fraction determination of fragmented and non-fragmented DNA of two cancer cell lines (however, PCR efficiencies of the primers for both types of DNA were not determined). Using our ddPCR strategy, a stable DII (see **equation E1**) of 1 was observed when analyzing non-fragmented DNA (gray bars) from both cell lines (60, 86, and 164 bp amplicons were used for determination of the quantity of short fragments while 302 bp was used for the determination of the quantity of the long fragment). These results suggested that a comparable amount of DNA was amplified regardless of the size of the tested fragments (Figures 2B1,2). When analyzing fragmented DNA (black bars), DII were inversely correlated with the size of amplicon selected as short fragments (164, 86, and 60 bp). Higher amounts of input DNA could be detected with the shorter amplicons. Meanwhile, as expected, similar DII were observed for the *KRAS* MT or WT sequences for both cell lines (Figures 2B1,B2, MT vs. WT bars). The percentage of *KRAS* MT DNA remained stable for the different analyses (Figures 2C1,C2), suggesting no detection bias of MT sequences over WT sequences. These results validate the pertinence of the developed ddPCR assays for the integrity analysis of both mutant and WT alleles.

### Detection of ctDNA Concentration in mCRC Patients Depends on the Amplicon Size Used for ddPCR Assays

**Figure 3:**
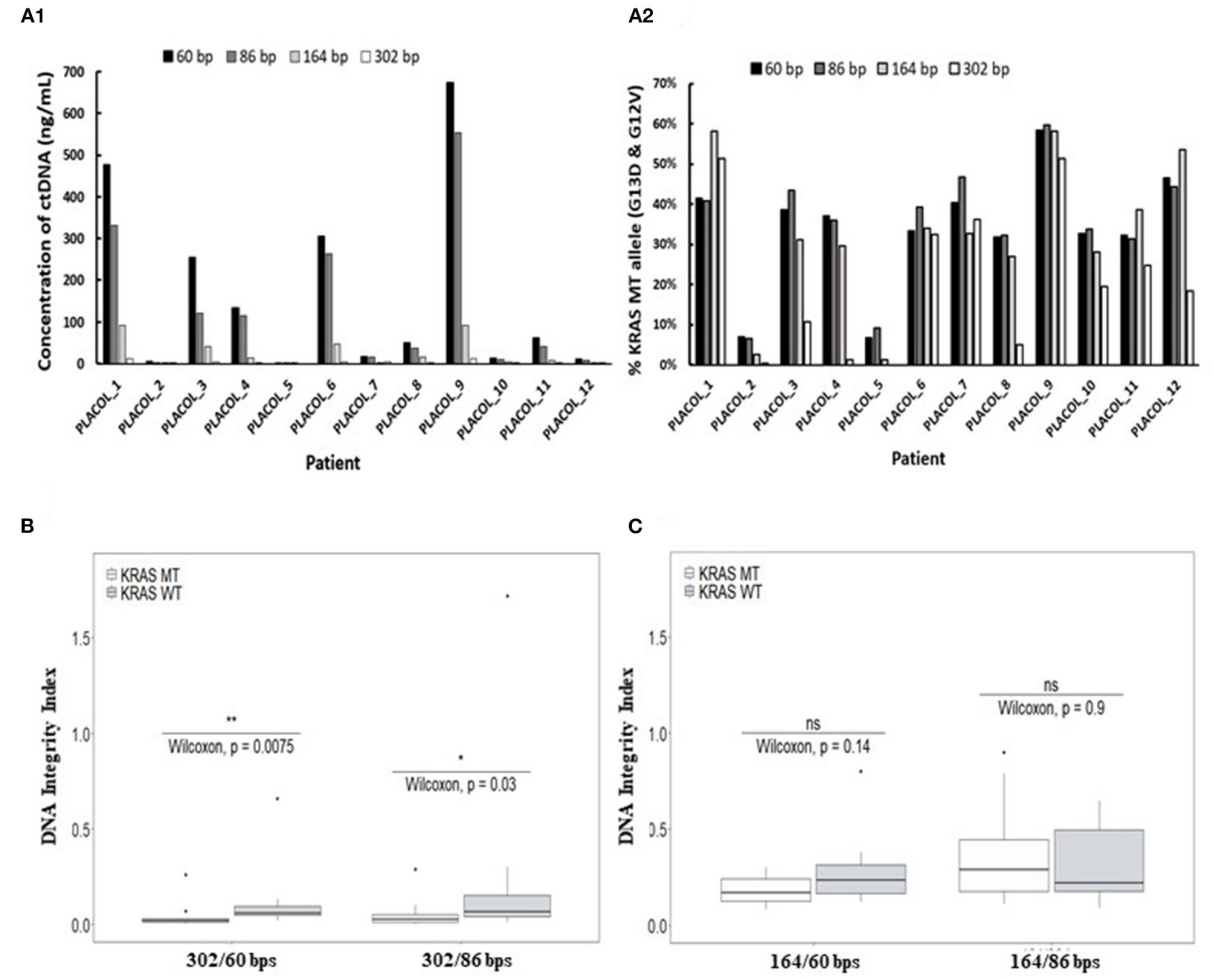
Characterization of ctDNA by droplet-based digital PCR using assays targeting increasing amplicon sizes. Concentration of plasma ctDNA **(A1)** and percentage of *KRAS*-mutated alleles **(A2)** in mCRC patients and DNA integrity indexes (DII) calculated with the use of 302 bp **(B)** or 164 bp amplicon **(C)** (corresponding to the long fragments) by ddPCR. *KRAS* WT and MT ddPCR duplex assays amplifying different sizes of fragments (60, 86, 164, and 302 bp) were used for measuring ctDNA concentration and percentage of *KRAS-*mutated alleles (**A1**,**A2**) for 11 mCRC patients (one patient excluded since no 302 bp MT fragments were detectable). DII were calculated based on the amplification of sequences with different lengths as described in the section **MATERIALS AND METHODS**. For DII calculation, 60 bp amplicons were used as the short fragments and either 302 bp **(B)** or 164 bp amplicon **(C)** as the long fragments. Mann–Whitney *U*-test was used for statistical significance analysis. *p < 0.05; **p < 0.01.

Using ddPCR assays in duplex format (as shown in Figure 2A), *KRAS* WT and MT DNA sequences could be precisely quantified, allowing to calculate the concentrations of each allele in the patient's samples. As shown in Figures 3A1,A2, ctDNA concentration and its proportion within total ccfDNA was linked to the targeted amplicon size used in the ddPCR integrity assay with smaller targeted amplicons leading to the detection of higher concentration of ctDNA (highlighted by the detection of *KRAS* MT alleles). Such results are in favor of a higher fragmentation of ctDNA and confirmed more efficient detection of ctDNA by ddPCR assays when targeting short amplicons.

### Determination of ctDNA Integrity in mCRC Patients by ddPCR

Droplet-based digital PCR integrity analysis targeting 60, 86, and 302 bp amplicons were performed in plasma ccfDNA of mCRC patients (PLACOL study, *n* = 12). As expected, a high fragmentation of ctDNA was observed in ccfDNA. DII results for the detection of MT allele are, respectively, 0.05 and 0.04 for the 302/86 and 302/60 bp amplicons. For the detection of WT allele, DII results are, respectively, 0.09 and 0.11 for 302/86 and 302/60. Significant differences between MT and WT alleles are observed (Wilcoxon test, *p* = 0.03 and 0.0075, respectively, for DII 302/86 and DII 302/60). Analysis of the MT fraction of ccfDNA highlighted an even stronger fragmentation with very low amount of 302 bp sequences detected compared with the WT sequence (Supplementary Table 5). The calculated DII of *KRAS* WT sequences was found significantly higher than the one calculated for *KRAS* MT sequences (Figure 3B, Wilcoxon test, *p* < 0.05). Since WT sequences can be released by both normal and tumor cells while MT sequences by tumor cells only, such results suggest a higher fragmentation of ctDNA as compared with the non-mutated fraction of ccfDNA. This was not observed when using 164 bp amplicon as long fragments for DII calculation (Figure 3C). Moreover, the concentration and fraction of ctDNA in patient samples detected by the *KRAS* MT allele appeared slightly higher when using 60 bp amplicon compared with the 86-bp one (Supplementary Figure 3). Based on these results, 60 bp amplicon was chosen as the short fragment and 302 bp amplicon as the long fragment to calculate ccfDNA and ctDNA DII in the following ddPCR experiments.

### Extraction Conditions Could Influence ccfDNA Integrity Profiles

**Figure 4:**
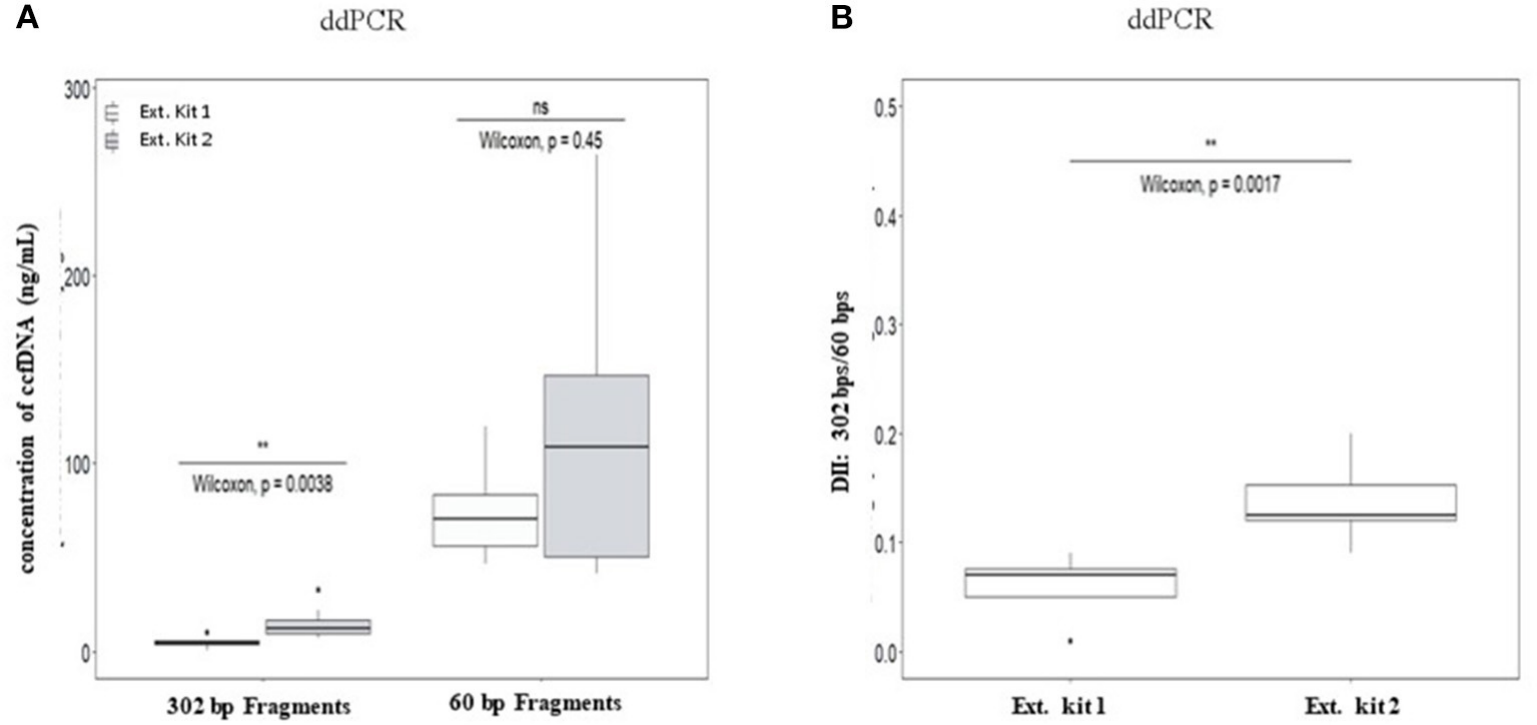
Comparison of the influence of ccfDNA extraction methods on the resulting ccfDNA fragment concentrations **(A)** and DNA integrity **(B)** by ddPCR. Plasma from 10 healthy individuals were used in this analysis (see workflow in Supplementary Figure 1). Maxwell RSC ccfDNA Plasma Kit (extraction kit 1) (Promega) and QIAmp® Circulating Nucleic Acid Kit (extraction kit 2) (Qiagen) were used for ccfDNA extraction. DII were calculated for ddPCR using 302 bp amplicon as the long fragment and 60 bp amplicon as the short fragment (*n* = 8, two individuals were excluded as no ccfDNA was available) **(B)**. Wilcoxon test was used for significance analysis. *p < 0.05; **p < 0.01; ***p < 0.001.

No significant differences of DII were observed between eight healthy plasma samples collected at the same time on K2-EDTA tube and cell-free DNA BCT° (STRECK) suggesting an absence of influence of the collection tube on ccfDNA fragmentation profile. (Supplementary Table 6; Supplementary Figure 4). We thus mainly focused on ccfDNA extraction methods on the ccfDNA quantity and DII in this study (workflow is described in Supplementary Figure 1). The results showed that a higher quantity of ccfDNA were extracted when using QIAmp® Circulating Nucleic Acid Kit (Ext.kit 2) as compared with the Maxwell RSC ccfDNA Plasma Kit (Ext.kit 1) (see Figure 4A) although the difference between the concentrations of 60 bp fragments was not statistically significant (*p* = 0.45). Moreover, DIIs calculated by ddPCR on the DNA extracted by the two methods appeared different (*p* = 0.0017) (Figure 4B). Such results confirmed the pertinence of preanalytic condition standardization not only for ccfDNA integrity determination but also in general for ccfDNA studies.

### Comparison of ccfDNA Integrity Index Between mCRC Patients and Healthy Individuals

**Figure 5:**
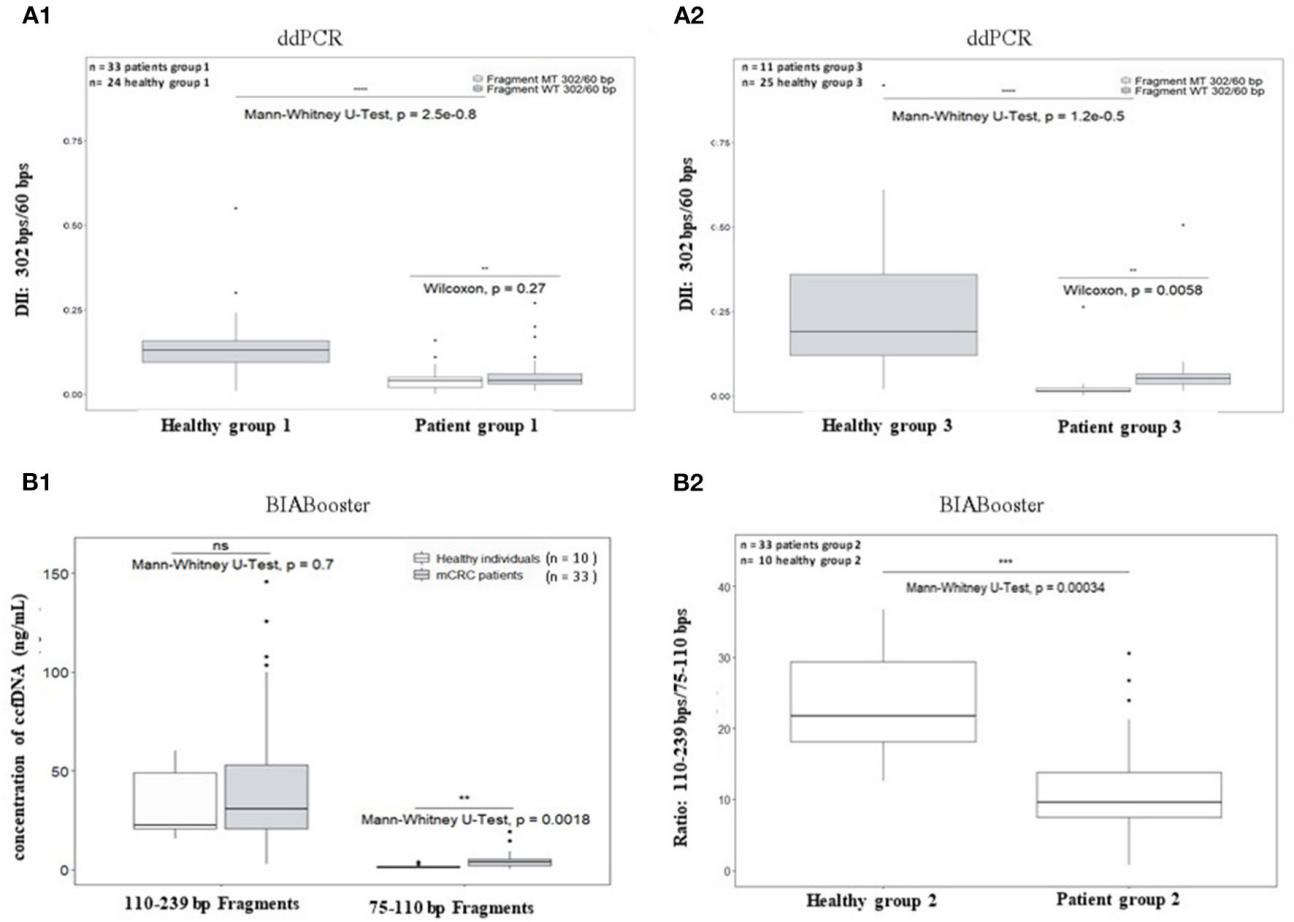
Comparison of plasma ccfDNA integrity and ccfDNA size profiling between healthy individuals and metastatic CRC patients. As ccfDNA from different patients' groups were prepared with different preanalytic conditions (blood collection tubes and ccfDNA extraction kits), DII comparative analysis was performed for each patient group samples using healthy subject samples collected and treated with the same procedure (see workflow Figure 2). To calculate DII by ddPCR, 302 bp amplicon was used as long fragment and 60 bp amplicon as short fragment (**A1**: AGEO RASANC cohort and **A2**: PLACOL cohort). Two samples (one from AGEO RASANC study and one from PLACOL study) have been excluded due to an absence of detection of droplets bearing DNA-mutated fragments of 302 bp or more. To investigate different size distributions by the BIAbooster System, fragments with size ranging from 110 to 239 bp were used as long fragments and fragments with size ranging from 75 to 110 bp as short fragments (**B1**,**B2**: AGEO RASANC cohort only). Mann–Whitney *U*-test was used for significance analysis. *p < 0.05; **p < 0.01; ***p < 0.001; ***p < 0.0005. Fragmentation of *KRAS* WT and MT alleles within ccfDNA for mCRC patients is shown in (**A1**,**A2**). Mutant-allele frequency determined with the DII assay was compared with the one determined previously using other assays for the RASANC samples [BPER NGS, Bachet et al. Ann Oncol. (25)] and PLACOL samples [ddPCR targeting 86 bp amplicon, Garlan et al. CCR. (26)]. Linear regression model is given in Supplementary Figure 6.

The DII of ccfDNA determined by ddPCR from both mCRC patients group were significantly lower than those from corresponding healthy plasma (Figure 5A1 for AGEO RASANC study, *p* = 2.5e−08 and Figure 5A2 for PLACOL study, *p* = 1.2e−05). However, when analyzing separately the DII of the *KRAS* WT and *KRAS* MT sequences in the two patient groups, a significant difference was observed for the PLACOL study (*p* = 0.0058) and not for the RASANC study (*p* = 0.27) (Figures 5A1,A2).

Circulating cell-free DNA size profiles from healthy individuals and mCRC patients was also compared using the BIAbooster System (Figures 5B1,B2). To better characterize small, fragmented DNA fraction, we characterized ccfDNA fragments between 75 and 110 bp (shorter than mono-nucleosomal DNA fragment lengths) and between 110 and 239 bp (comprising expected mono-nucleosomal DNA fragment lengths (140–180 bp). However, since the PLACOL cohort was extracted using the QIAamp Circulating Nucleic Acid Kit that implies the use of RNA carrier that can compromise BIAbooster fragments analysis, only the sample from AGEO RASANC cohort were analyzed here. As shown in Figure 5B1, a higher quantity of short fragments (75–110 bp) was observed in mCRC patients than in healthy individuals (Figure 5B1) and no difference was found for 110–239 bp fragments. Furthermore, similar DII differences were observed by ddPCR. The ratio of the quantity of fragments between 110 and 239 bp and 75 and 110 bp was also significantly different between mCRC patients and healthy individuals (Figure 5B2). These results further reinforced the potentiality of using ccfDNA integrity and its size profiling as a diagnostic or follow-up tool for mCRC patients.

## Discussion

Up to now, most strategies of ctDNA detection are based on the targeting of tumor-specific mutations which implies the conception of a numerous assays or large NGS gene panels to cover a maximum of possible mutations. The use of epigenetic alterations such as hypermethylated sequences has been recently described in mCRC (27) but remains cancer specific. The characterization of DNA integrity has long been suggested as a potentially “universal” cancer marker (6). However, contradictory results have limited researchers' interest, which drew our attention on the importance of standardization.

The necessary normalization of the preanalytical conditions has been highlighted (28–30) and is also central to large research/network initiatives (31). As different clinical studies could present different preanalytical treatments, it is necessary to investigate the potential impact of preanalytical methods on the efficiency of purification of the different sizes of DNA fragments.

Several studies have recalled the impact of the collection tube on the ccfDNA integrity (32–34). The collection tubes mostly differs on their content in preservative agents that prevent blood cell hemolysis (35). We did not observe such impact in our study (Supplementary Figure 4). This could be linked to the low number of healthy individuals studied. It could also be explain by the fact that the EDTA collection tubes were processed early after collection within the PLACOL study (26), limiting unwanted DNA fragmentation. Indeed, it has been shown that when tubes are not handled adequately, the genomic DNA coming from the blood cell could be released. This genomic DNA could both dilute the ccfDNA tumor fraction and disturb its fragmentation profile. The increase of multicentric clinical studies and the centralization of the analysis platforms could cause uncontrolled delays between blood collection and plasma separation. For these reasons, several ccfDNA preservative tubes have been developed, such as the BCT Streck tubes used in this study (36), that could become a reference collection tube dedicated to liquid biopsy (30).

Recent studies that compared extraction methods for the isolation of ccfDNA from plasma samples have concluded that they can affect both ccfDNA yield and fragmentation (32, 37). Our work confirmed these results showing significant differences both in terms of fragmentation and ccfDNA concentration.

Originator studies that have analyzed the DNA integrity in cancers, relied on the qPCR analysis of repetitive unspecific DNA sequences (ALU, LINE1) (16). Recent works however, have been developed to specifically measure integrity indexes while targeting cancer-specific mutated sequences by qPCR (23). These studies are nevertheless limited by the sensitivity and accuracy of basic bulk PCR techniques. Emerging methods based on droplet-based digital PCR could reach both high sensitivity and accuracy (38, 39) especially when following the Digital MIQE Guidelines (40). Moreover, innovative procedures have also been developed for fragment-based highly sensitive detection (19, 24). Our results demonstrated, using two independent strategies, a significant difference of ccfDNA fragmentation in plasma of mCRC patients compared with healthy subjects. Moreover, when specifically targeting the fraction of ccfDNA bearing cancer-specific mutations (and thus discriminating ctDNA) by ddPCR, significant differences were also observed between DNA bearing cancer-specific mutation(s) and non-mutated DNA in the patients from the PLACOL cohort. Such results confirmed the high fragmentation of ctDNA (with most fragments below 150 bp as highlighted by both strategies used), which is consistent with other studies (18, 41, 42). BIAbooster system provided complementary data to the ddPCR assay, evoking that a major part of the ctDNA could even be smaller than 110 bp (Figure 5B1; Supplementary Figure 5). The two methods lead to complementary information. Indeed digital-PCR assay quantifies amplifiable DNA fragments larger than a chosen size (i.e., if a 60-bp amplicon is amplified, detected DNA fragments be larger than 60 bp including the targeted sequence) whereas BIAbooster system determines the quantity of DNA fragments within a given size range (for example, between 75 and 110 bp).

In addition to pursuing the development of DII measurement of ccfDNA as a universal cancer marker, such results have strong implications with regard to future assay design. Indeed, we showed that assay targeting short amplicons allowed more sensitive and precise quantification of ctDNA, which is especially relevant for the analysis of low concentrated plasma samples. Interestingly, using IntPlex, Moulière et al. demonstrated that the targeting of small fragments within ccfDNA permits to increase the efficiency of the detection of ctDNA (23). In a more recent work, they also improved the detection of ctDNA by analyzing the shorter fragments after adding an either *in vitro* or *in silico* size selection step (43). Xiao Liu et al. also enhanced the detection of pancreatic cancer by developing a new single-strand library preparation and hybrid-capture-based ccfDNA sequencing (SLHC-seq) to analyze small ccfDNA fragments (<100 bp) (44).

In conclusion, with the combined consideration of the importance of the standardization of preanalytical and analytical procedures as well as the increase of the sensibility and accuracy of analytical methods, the measure of DNA integrity should be an interesting parameter to develop new clinical biomarker in oncology. In particular, even if differences have been observed between sample preparation procedures, the crucial point appeared to ensure method consistency within a single study. Indeed, even if the ext.kit 2 may allow for highest purification of small DNA fragments (and thus probably of tumoral DNA enriched fraction) as compared with ext.kit 1, it also seems to lead to strongest variations in extraction efficiency (see Figure 4).Further analyses, involving a larger number of samples are required to allow for definitive conclusions. Moreover, the described procedures will allow conducting similar studies on newly developed ccfDNA extraction kits. The optimizations of the described ddPCR method could involve the development of multiplex assay that allows the measurement of different fragment sizes in a single pot. Yet, the limited number of patients in our study also requires further investigations. Another limitation is that we limited our analysis to patients with mCRC. Moreover, to fully validate the DII as cancer biomarker, further investigations about the cfDNA biology, in particular about the potential influence of aging on cfDNA fragmentation could be of interest. In order to assess its role in a more general setting, further studies should involve various cancer stages as well as other primary tumors. Moreover, investigating the processes leading to the observed differences of fragmentation profiles between tumor and healthy cells derived DNA would greatly contribute to the use of DII measurement as a universal cancer marker.

## Data Availability Statement

The datasets presented in this study can be found in online repositories. The names of the repository/repositories and accession number(s) can be found in the article/Supplementary Material.

## Ethics Statement

The studies involving human participants were reviewed and approved by NCT02502656 (AGEO RASANC). The patients/participants provided their written informed consent to participate in this study.

## Author Contributions

All authors listed have made a substantial, direct and intellectual contribution to the work, and approved it for publication.

## Conflict of Interest

VT: Honoraria from Raindance Technologies and Boerhinger Ingehleim; cofounder emulseo; board emulseo. JT: Honoraria from Merck, Amgen, Roche, Pierre Fabre, MSD, Sanofi, and Lilly, Servier, and Astra-Zeneca. AZ: consulting and/or advisory boards for: Roche, Merck Serono, Amgen, Sanofi, and Lilly. PL-P: Honoraria and board: Amgen, Merck-Serono, Boehringer Ingelheim, Sanofi, Roche, and Lilly. HB: Honoraria Astra-Zeneca, BMS, MSD. JT: Honoraria from Merck, AMGEN, ROCHE, SIRTEX, BAXALTA, SANOFI, Lilly, and Servier. AZ: Consulting and/or advisory boards for Roche, Merck Serono, Amgen, Sanofi, and Lilly. GP, LP, and VG: Eurofins-Biomnis employee. FGa: Bio-Rad employee. AB and FGi were employed by company ADELIS. The remaining authors declare that the research was conducted in the absence of any commercial or financial relationships that could be construed as a potential conflict of interest.

## Abbreviations

bp: base pairs
ccfDNA: circulating cell-free DNA
ctDNA: circulating tumor DNA
ddPCR: droplet-based digital PCR
DII: DNA Integrity Index
qPCR: quantitative PCR
mCRC: metastatic colorectal cancer
MT: mutant
NGS: next-generation sequencing
nt: nucleotide
WT: wild-type
*KRAS*: v-Ki-ras2 Kirsten rat sarcoma viral oncogene homolog.
