# Supplementary material for: Characterization of Plasma Cell-Free DNA Integrity Using Droplet-Based Digital PCR: Toward the Development of Circulating Tumor DNA-Dedicated Assays
Source: Front Oncol. 2021 May 6;11:639675. doi: 10.3389/fonc.2021.639675 (PMC8174096; doi:10.3389/fonc.2021.639675)
Supplement: Supplementary file 1 [file Data_Sheet_1.docx]

**Supplemental Methods**

**Title: Characterization of plasma cell-free DNA integrity using picoliter-droplet digital PCR: towards the development of circulating tumor DNA - dedicated assay**

**Running head: Circulating tumor DNA integrity characterization**

**List of authors:** Geoffroy Poulet^1,2,#^, Fanny Garlan^1,#^, Sonia Garrigou^1^, Eleonora Zonta^1^, Leonor Benhaim^1,3^, Marie-Jennifer Carrillon^1^, Audrey Didelot^1^, Delphine Le Corre^1^, Claire Mulot^1,9^, Philippe Nizard^1^, Frederic Ginot^4^, Audrey Boutonnet-Rodat^4^, Helene Blons^1,5^, Jean-Baptiste Bachet^1,6,7,8^, Julien Taïeb^1,5,8^, Aziz Zaanan^1,5^, Vanna Geromel^2^, Laurence Pellegrina^2^, Pierre Laurent-Puig^1,5^, Shu-Fang Wang-Renault^1,*^, Valerie Taly^1,*^.


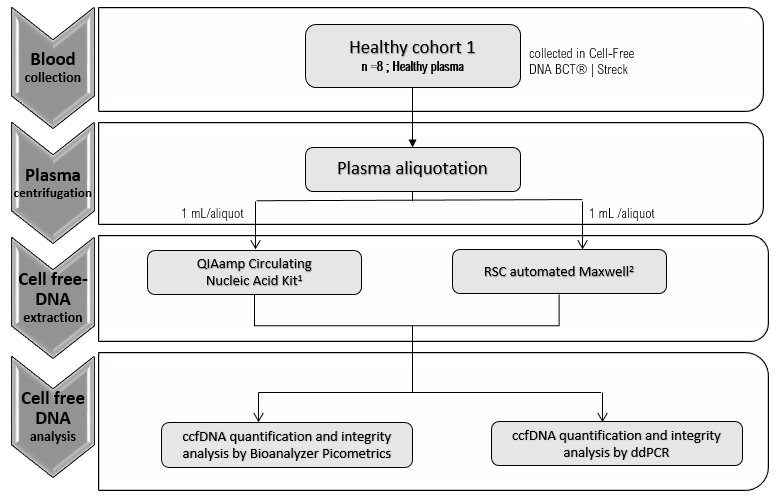


***Supplementary Figure 1: Workflow of preanalytical study***

Schematic workflow of healthy plasma used in this study to determine the impact of the extraction method on the ccfDNA integrity. 2 kits ccfDNA purification are compared: (1) RSC Automated Maxwell (Promega) using magnetic beads and (2) QIAmp® Circulating Nucleic Acid Kit (Qiagen SA) using purification column.


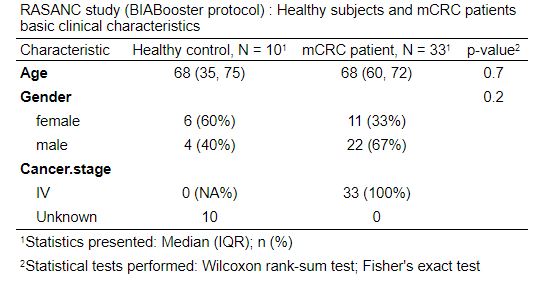

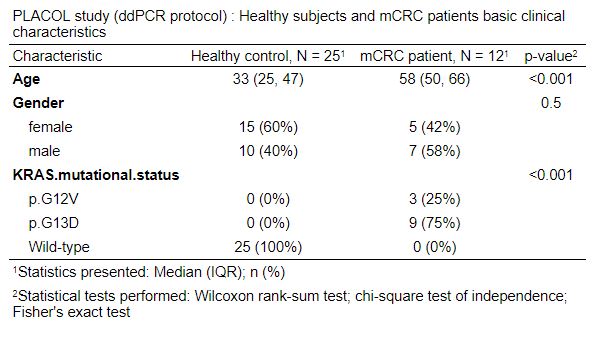

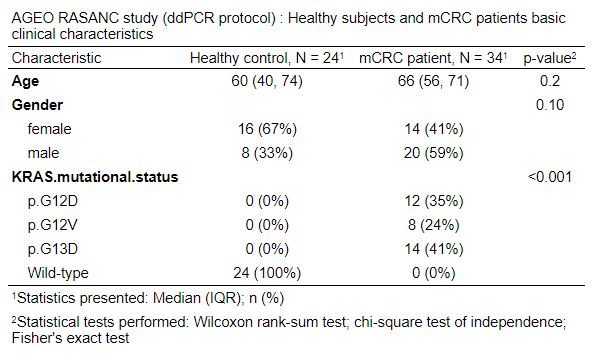


**Supplementary Table 1: Basic clinical characteristics of healthy subjects and mCRC patients included in the study.**

***Supplementary Table 2: Experimental parameters used on S220 Covaris Focused-Ultrasonicator***

| **Parameters** | **Fragment (700 bps)** |
| --- | --- |
| Peak Intensity | 105 |
| Dusty force | 10 |
| Cycle/burst | 200 |
| Time (s) | 80 |
| Temperature Range (°C) | 9-12 |

***Plasma ccfDNA preparation***

Every blood sample has followed two centrifugation steps: the first one at low-speed (1600g, 20 min) and the second one at high-speed (15,000 rpm, 15 min). Plasma samples have been stored at -80°C for 3-5 years (PLACOL/RASANC study) and < 9 months for healthy plasmas. Every ccfDNA was extracted from the plasma with the use of the Maxwell RSC ccfDNA Plasma kit (Promega) according to the manufacturer’s instructions or using the QIAmp® Circulating Nucleic Acid Kit (Qiagen) and eluted in both cases in 50 µL of elution buffer.

***Statistical analysis***

Statistical analysis was performed using R software (version 3.5.3). For the determination of the optimized DNA integrity index and a strict comparison between the wild-type alleles (for healthy patient & mCRC patients) and the mutant alleles (mCRC patients only), a paired nonparametric Wilcoxon was used. For the analysis of the DNA integrity index difference between RSC automated Maxwell and QIAmp® Circulating Nucleic Acid Kit after DNA extraction from plasma of healthy subjects, a paired nonparametric Wilcoxon test was used. For the analysis of the DNA integrity index differences between Healthy plasmas and mCRC plasmas, unpaired nonparametric Mann-Whitney U-test was performed.

***
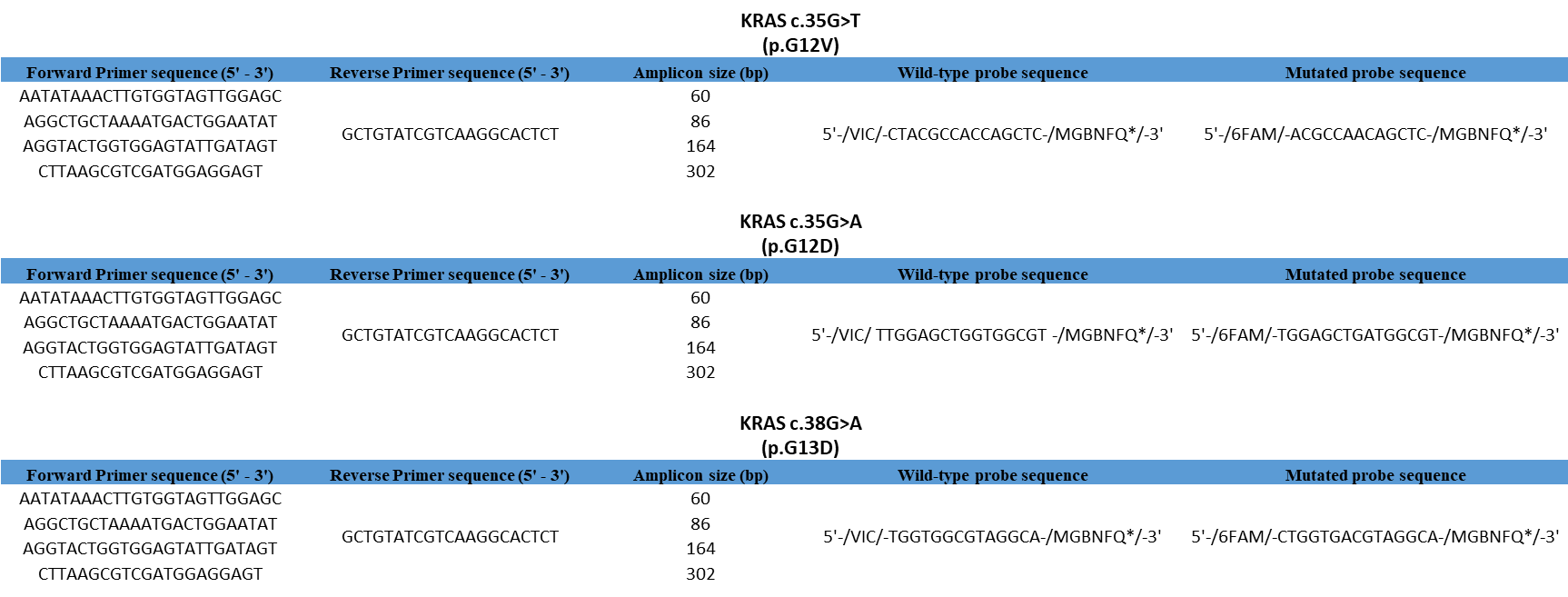
Supplementary Table 3: Primers and probes sequences for the different digital PCR assays***

***Supplementary Table 4: PCR Programs***

| PCR Step | Temp (°C) | 60 & 86 bp | 164 bp | 302 bp | Cycle | Ramp rate (°C/min) |
| --- | --- | --- | --- | --- | --- | --- |
| Enzyme activation | *50* | *2’* | | | *1* |  |
| DNA denaturation | *95* | *10’* | | | *1* | *0.6* |
| Primers & Probe hybridization and elongation | *95*  *64* | *15’’*  *45’’* | *15’’*  *1’* | *15’’*  *1’ 15’’* | *45* | *0.6*  *0.6* |
| Signal Stabilization | *98*  *12* | *10’*  *10’* | | | *1* | *0.6* |

***Table 5: DII obtained in mCRC patients using different KRAS p.G13D and G12V assays.*** *ddPCR analysis of this sample did not show any 302pb mutated fragments. Consequently ratios involving theses fragments can’t be calculated precisely and were estimated (see main text, Materials and methods section).

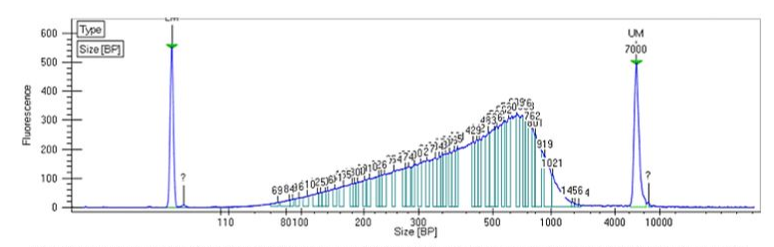


***Supplementary Figure 2: Size distribution of Ultrasonicator-fragmented genomic DNA confirmed using the Caliper system.*** One µg of genomic DNA in 130µL water was pipeted in an appropriate vessel according to manufacturer’s instruction. Acoustic bursts were delivered to samples with a peak incident power of 130W for 80 seconds, and a duty factor of 5%, thus tuning shearing forces to obtain a main pick located between 500 and 1000 bps. The size distribution of fragments was confirmed using the Caliper system (Lab chip GX/GXII Microfluidic system (Perkin-Elmer), DNA assay S or 5K reagent kit (Perkin-Elmer)).


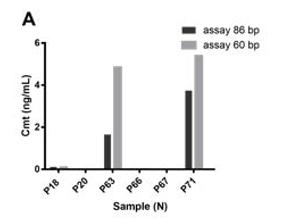

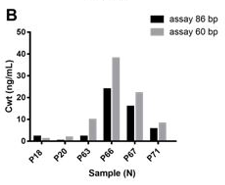

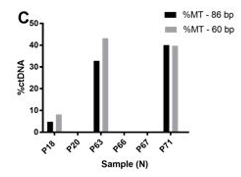


***Supplementary Figure 3: Concentration of circulating mutant (MT) (A) and wild-type (WT) (B) DNA and percentage of KRAS mutated alleles in respective samples (C).*** These patients (PLACOL patient group) presented very low amounts of plasma tumor DNA, however a higher concentration of both MT and WT DNA was detected when using the 60 bp assay for ctDNA determination.

**Table 6: Data summary - Comparison of calculated DII between 8 ccfDNA samples of healthy subjects collected in EDTA tubes or cell-free DNA BCT° tubes (STRECK) and extracted in both cases manually using QIAamp Circulating Nucleic Acid Kit.**


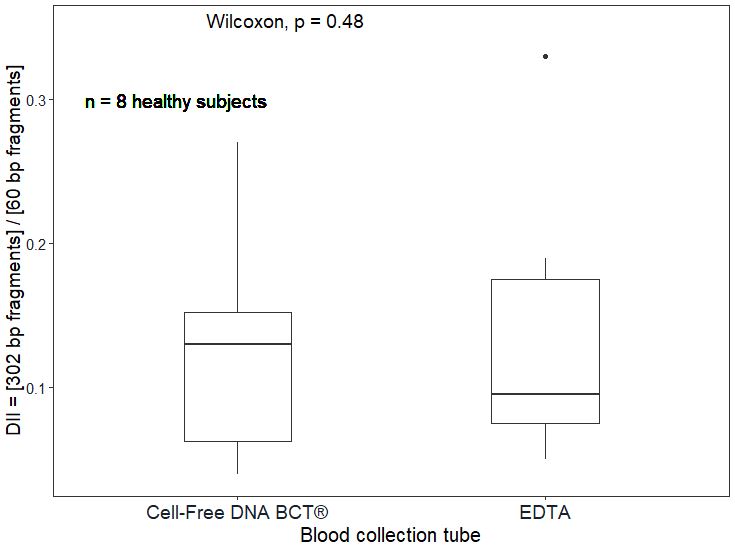


**Supplementary Figure 4: Comparison of calculated DII between eight ccfDNA samples from healthy subjects collected in EDTA tubes or cell-free DNA BCT° (STRECK) and extracted in both cases manually using QIAamp Circulating Nucleic Acid Kit.**


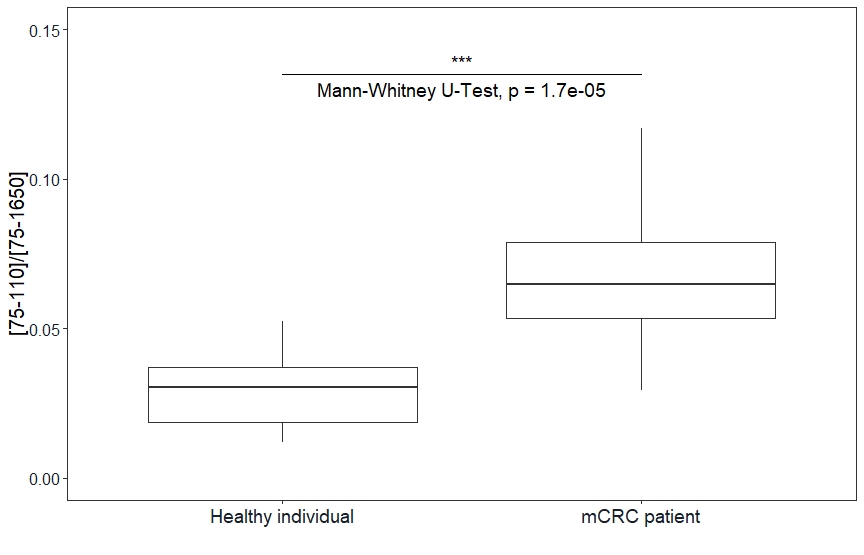

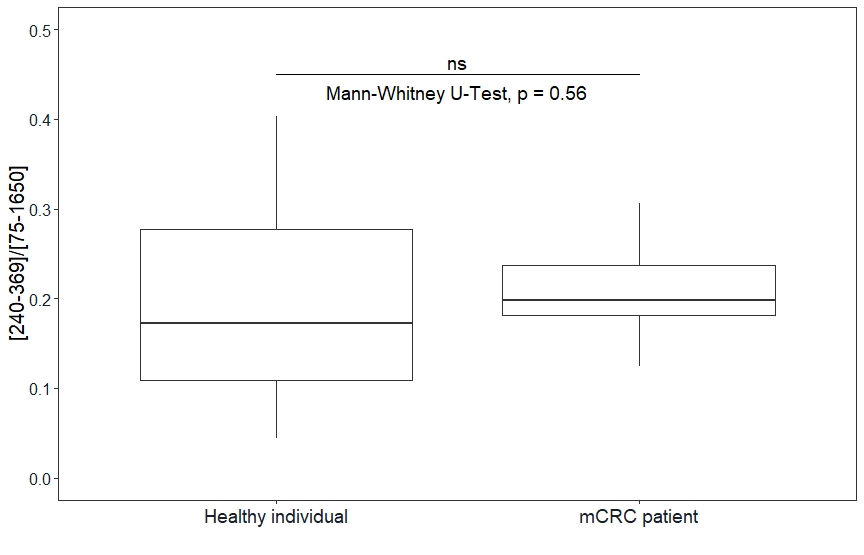

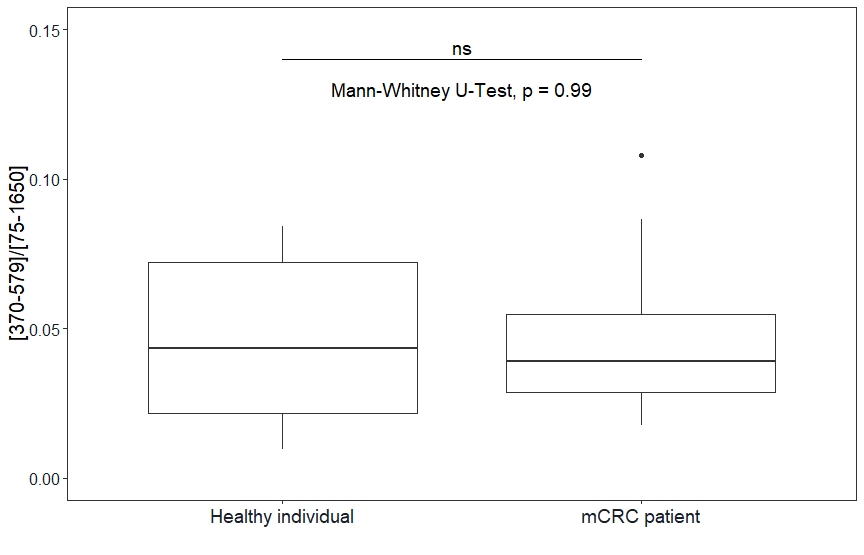

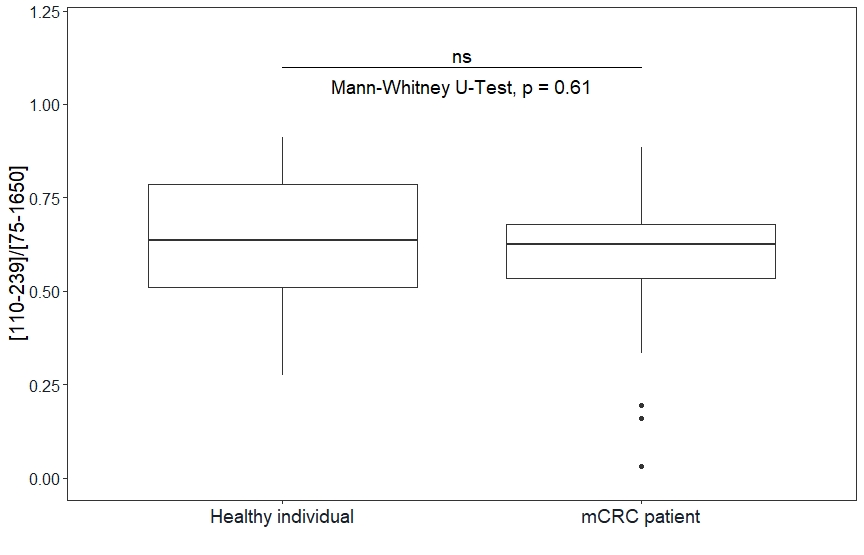


**A**

**B**

**C**

**D**

***Supplementary Figure 5: Concentrations of DNA for the different analysed fragment size ranges (ie. [75-110], [110-239], [240-369] and [370-579] bps) for plasma samples of healthy subjects (n= 10) and metastatic CRC patients (n=33) (AGEO RASANC study) extracted by RSC automated Maxwell.*** Significant differences are observed (Mann-Whitney, p-value = 1.7*10^-5^) for fragments [75-110] bps between healthy subjects and mCRC patients. Differences for other fragment ranges were not significant. We observed an equal repartition of fragment sizes between [110-579] bps after a RSC automated Maxwell extraction between the 2 types of plasma samples.

**
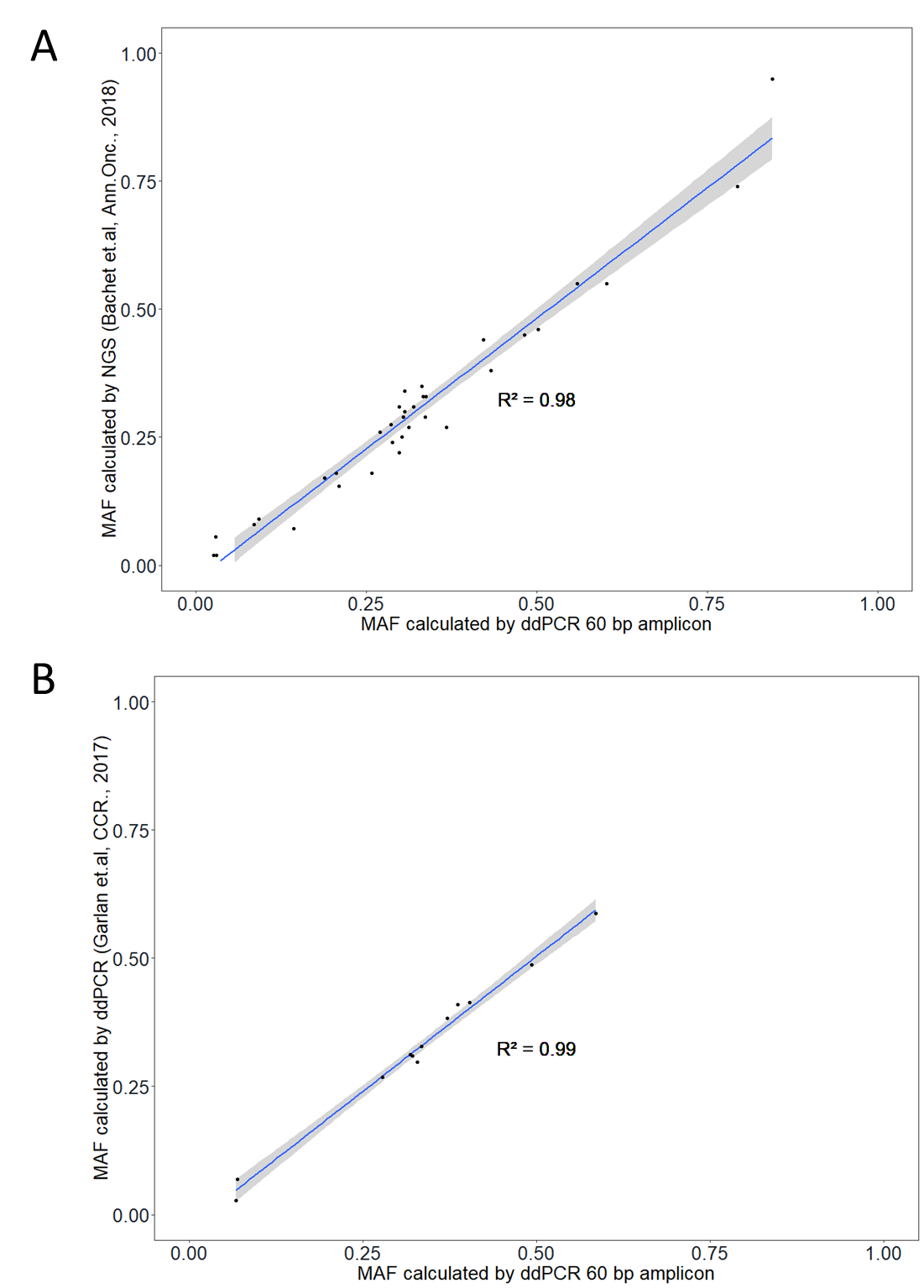
**

**Supplementary Figure 6:** **Comparison of mutant *KRAS* allelic fraction determined by the newly developed assay targeting 60bp amplicon or other methods. A) Pearson correlation with NGS data (Bachet *et al.* 2018, Ann. Onc, 2018); B) Pearson correlation with duplex ddPCR targeting 86 bp amplicon (Garlan *et al*., CCR, 2017).**

**Bibliography**

Bachet JB, Bouché O, Taieb J, Dubreuil O, Garcia ML, Meurisse A, et al. RAS mutation analysis in circulating tumor DNA from patients with metastatic colorectal cancer: the AGEO RASANC prospective multicenter study. Ann Oncol. 2018;29:1211–9.

Garlan F, Laurent-Puig P, Blons H, Taly V. Early Evaluation of Circulating Tumor DNA as Marker of Therapeutic Efficacy in Metastatic Colorectal Cancer Patients (PLACOL Study). Clinical Cancer Research. 2017;23:5416–25.
